# Intein mediated hyper-production of authentic human basic fibroblast growth factor in *Escherichia coli*

**DOI:** 10.1038/srep33948

**Published:** 2016-09-22

**Authors:** Keith W. Y. Kwong, T. Sivakumar, W. K. R. Wong

**Affiliations:** 1 Division of Life Science, The Hong Kong University of Science and Technology, Clear Water Bay, Kowloon, Hong Kong, China

## Abstract

Human basic fibroblast growth factor is a functionally versatile but very expensive polypeptide. In this communication, employing a novel amplification method for the target gene and genetic optimization of a previously engineered expression construct, pWK3R, together with a refined fed-batch fermentation protocol, we report an achievement of a phenomenal yield of 610 mg/L of the 146 aa authentic human basic fibroblast growth factor (bFGF) in *Escherichia coli*. Construct pWK3R was first modified to form plasmid pWK311ROmpAd, which was devoid of the *ompA* leader sequence and possessed two copies of a DNA segment encoding a fusion product comprising an intein, *Saccharomyces cerevisiae* vascular membrane ATPase (VMA), and bFGF. When *E. coli* transformant JM101 [pWK311ROmpAd] was cultivated using the refined fed-batch fermentation protocol, superb expression resulting in a total yield of 610 mg/L of bFGF was detected. Despite existing in high levels, the bFGF remained to be soluble and highly bioactive.

Cost-effective production of recombinant proteins is a prerequisite for the widespread availability of the products on the market. Human basic fibroblast growth factor, notwithstanding a versatile protein shown to play important functions in various physiological processes including angiogenesis, wound healing and chondrogenesis^1^,^2^,^3^, has not been commonly applied as expected. Authentic human basic fibroblast growth factor (bFGF) is a 16.5 kDa protein comprising 146 aa residues^1^. However, essentially only structural analogs of bFGF of various molecular sizes are available for commercial applications^4^,^5^,^6^,^7^. The reason is probably due to the use of conventional cloning methods, which are unable to establish a cost-effective processing protocol, to result in bFGF. Thus, bFGF has not been commonly available for skin care or therapeutic applications. Incredibly, however, despite being unauthentic, bFGF analogs are sold at extremely high prices, ranging from US$ 1,300 to US$ 2,000 per mg^8^. Thus, only cost-effective availability of bFGF on the market may help lower its unreasonably high prices.

Our laboratory has been involved in the engineering of various recombinant host systems for efficient expression of valuable proteins^9^,^10^,^11^,^12^,^13^,^14^,^15^,^16^,^17^. Recently, with *Bacillus subtilis* as the host, we have been successful in achieving secretory expression of fully bioactive bFGF^10^. Later on, exploiting the self-cleavable ability of protein introns, which are also known as “inteins”^18^,^19^,^20^,^21^, we have engineered intein-mediated expression constructs employing *E. coli* as the host. Mediated by the coding sequence for an intein, *Saccharomyces cerevisiae* vascular membrane ATPase (VMA), we have engineered an efficient protein expression construct, pWK3R, to successfully co-express bFGF and human epidermal growth factor (EGF) as bioactive and authentic products^9^. The same expression approach has also been employed to efficiently co-express widely dissimilar recombinant proteins^11^. Since the target proteins are present in both the cytoplasm and growth media of the expression systems concerned, the utilization of all compartments of the culture for storage may effectively enhance the overall yields of the desired proteins^9^,^11^.

In this communication, through genetic optimization of a VMA-mediated expression construct, pWK3R, in which the *ompA* leader sequence was deleted and the coding sequence for a VMA-bFGF fusion product was increased to two copies, an efficient expression derivative, pWK311ROmpAd, was constructed. When its *E. coli* transformant, JM101 [pWK311ROmpAd], was cultivated using a refined fed-batch fermentation protocol, a phenomenal yield of 610 mg/L of bFGF, which was shown to be 7.5 times as high as that obtained from JM101 [pWK3R] reported previously^9^, was attained.

## Results

### Strategy for enhancing expression of recombinant bFGF

**Figure 1:**
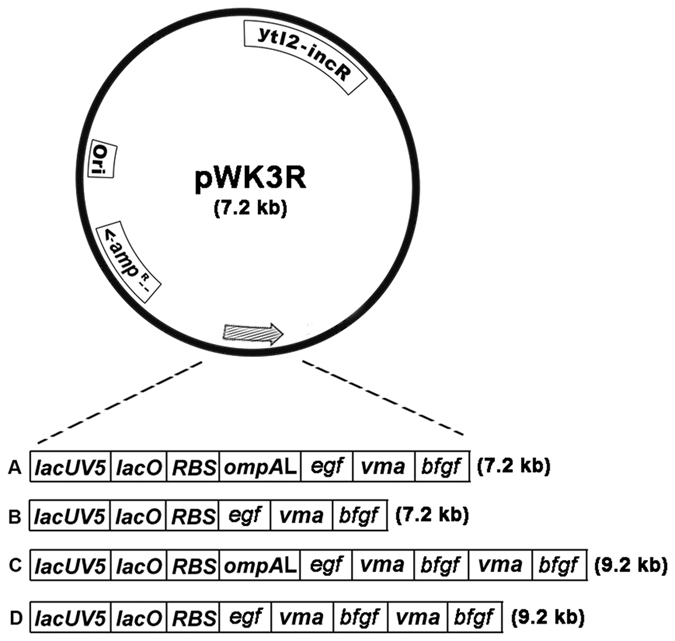
Schematic representation of DNA constructs expressing bFGF. The top diagram shows the parental vector pWK3R, with the hatched arrow representing the genetic elements responsible for bFGF expression. The four constructs: (**A**) pWK3R, and its three derivatives: (**B**) pWK3ROmpAd, (**C**) pWK311R, and (**D**) pWK311ROmpAd, together with the genetic elements forming their own hatched arrows are specified underneath. Symbols for the components shown in pWK3R and its three derivatives are: *ori* = origin of replication in *E. coli*; *amp*^R^ = structural gene conferring resistance to ampicillin; ytl2-incR* = ytl2-incR* system for plasmid stability of *Salmonella typhimurium*; *bfgf* = *bfgf* gene; *egf* = *egf* gene; *lacUV5* = lacUV5 promoter; *lacO* = lac operator; *RBS* = consensus ribosomal binding site; *ompA*L = *ompA* leader sequence; *vma* = VMA coding sequence. Arrow indicates the direction of transcription. Information in parenthesis indicates the plasmid size.

Previously, employing a human epidermal growth factor (EGF) excretion plasmid, pWKW2^13^,^14^,^15^, and the coding sequence for an intein, *Saccharomyces cerevisiae* vascular membrane ATPase (VMA), we engineered an expression construct, pWK3R (Fig. 1)^9^, to achieve co-expression of authentic EGF and human basic fibroblast growth factor (bFGF) in *E. coli*^9^. VMA, which was envisaged acting as a self-separable locking apparatus^9^,^19^, could not only mediate co-expression of EGF and bFGF, but also facilitate auto-cleavages of both recombinant EGF and bFGF from the EGF-VMA-bFGF precursor and intermediates^9^. In this study, we attempted to largely enhance the expression of bFGF through a systematic approach involving two steps: first, by genetic modifications of pWK3R to delete the *ompA* leader sequence, and applying a novel amplification method, to achieve a two-fold increase in the copy number of the *bfgf* gene; and second, to further enhance the productivity of an optimized transformant expressing bFGF, employing a refined fed-batch fermentation protocol.

### Engineering of DNA constructs expressing bFGF

Essentially, two different genetic modifications were performed on pWK3R (Fig. 1A) to result in three plasmid derivatives: pWK3ROmpAd, pWK311R and pWK311ROmpAd (Fig. 1). In constructs pWK3ROmpAd and pWK311ROmpAd, the *ompA* leader originally carried on pWK3R was deleted. The major difference between these two derivatives was that pWK311ROmpAd (Fig. 1D) carried two copies of a DNA fusion product formed between the VMA coding sequence (*vma*) and the *bfgf* gene, whereas pWK3ROmpAd (Fig. 1B) had only one copy of the DNA fusion concerned. On the other hand, similar to pWK311ROmpAd (Fig. 1D), pWK311R (Fig. 1C) was also modified to contain two copies of the mentioned DNA fusion. However, it retained the *ompA* leader, thus enabling also secretory, in addition to intracellular, production of both EGF and bFGF.

### Time course studies

Expression of bFGF in *E. coli* JM101 transformants harboring the four plasmids: pWK3R, pWK3ROmpAd, pWK311R and pWK311ROmpAd (Fig. 1) was first compared in shake flasks under IPTG induction. Western blot analysis of culture samples revealed that in addition to JM101 [pWK3R], which was capable of producing bFGF as reported previously^9^, the other three transformants were also able to express bFGF as a fully processed protein (Table 1). The results support the conclusion that the precursor/intermediate products, including EGF-VMA-bFGF, OmpA-EGF-VMA-bFGF-VMA-bFGF, OmpA-EGF-VMA-bFGF and EGF-VMA-bFGF-VMA-bFGF, which were initially expressed in the three transformants harboring the construct derivatives, were able to undergo auto-cleavable activities to yield bFGF (Table 1), with the developments similar to that observed previously in transformant JM101 [pWK3R]^9^.

**Figure 2:**
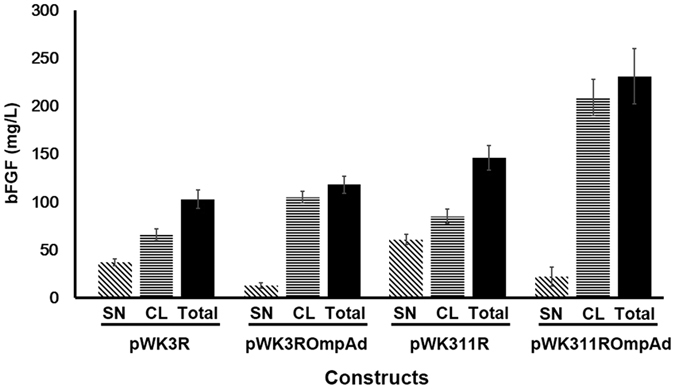
Quantification of bFGF detected in fractionated samples of *E. coli* transformants harboring various constructs. *E. coli* transformants harboring the four constructs as indicated beneath the x-axis were grown in shake flasks as described in Methods. The cultures were fractionated into culture supernatant (SN) and cell lysate (CL) samples, which were then assayed by western blot analysis, followed by quantification of bFGF. Each experiment was repeated three times and standard error bars are shown.

Despite employing the same regulatory controls and intein, VMA, the four constructs resulted in noticeable difference in the level of bFGF expression (Fig. 2). The results revealed that the three plasmid derivatives were more efficient than pWK3R in expressing bFGF (Fig. 2). The improvements were likely attributable to a two-fold increase in the copy number of the *bfgf* gene, in particular when constructs pWK311R and pWK311ROmpAd (Fig. 1) were considered. Their levels of bFGF expression were significantly higher than that resulting from pWK3R (Fig. 2).

On the other hand, deletion of the OmpA signal peptide from the precursor products was notably beneficial to the overall expression of bFGF. Without the signal peptide, essentially all resources for bFGF production would be confined in the cytoplasm, as in the case of JM101 [pWK3ROmpAd] and JM101 [pWK311ROmpAd], thereby providing higher yields of bFGF than their respective counterparts, JM101 [pWK3R] and JM101 [pWK311R] (Fig. 2), in which their constructs contained the *ompA* leader (Fig. 1).

### Primary structure of bFGF expressed in *E. coli* tranformants harboring the four constructs

The primary structure of bFGF purified from the culture media and cell lysates of *E. coli* transformants harboring pWK3R, pWK3ROmpAd, pWK311R and pWK311ROmpAd was determined by liquid chromatography tandem mass spectrometry as previously reported^9^. Sequencing results confirmed that both the supernatant and cytoplasmic bFGF samples purified from the cultures of the four transformants contained the correct N- and C-termini (Table 1), supporting the conclusion that the bFGF samples comprised 146 aa residues, with the same composition as that of native bFGF^1^.

Another noteworthy point concerns the homogeneity of bFGF resulting from the expression of constructs pWK311R (Fig. 1C) and pWK311ROmpAd (Fig. 1D), each of which possessed two copies of the *bfgf* gene fused with two copies of *vma* to form a DNA fusion: *vma*/*bfgf*/*vma*/*bfgf*. Although the leftward *bfgf* gene in both of the constructs concerned was flanked by two copies of *vma*, aa composition analysis revealed the presence of a homogeneous set of tryptic peptides derived from the hydrolysis of bFGF (Table 1). The results support the conclusion that irrespective of whether bFGF is fused at either the N- or C- terminus of VMA, intriguingly, the cleavage at either junction is precisely accomplished as desired to yield the authentic product.

### Activities of bFGF expressed by constructs pWK3ROmpAd, pWK311R and pWK311ROmpAd

**Figure 3:**
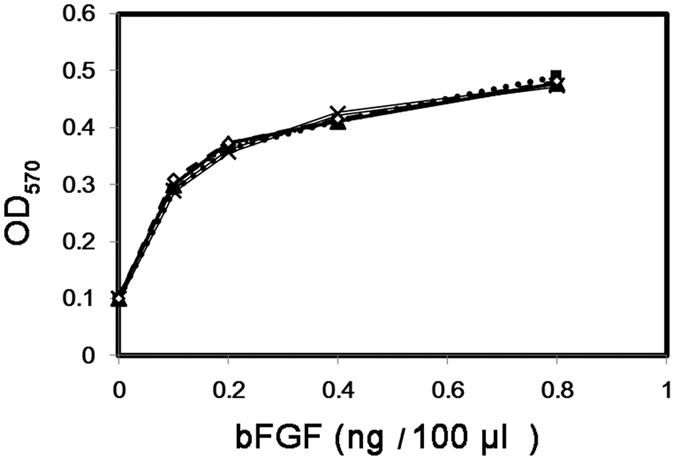
Bioassays of bFGF produced by the four transformants. Samples of bFGF were purified from IPTG induced cultures of JM101[pWK3ROmpAd], JM101[pWK311R] and JM101[pWK311ROmpAd]. The assays for mitogenic effects of bFGF on the proliferation of BALB/C 3T3 fibroblast cells were described in Methods. The bioactivities of standard bFGF (==X==), JM101[pWK3ROmpAd] (^___^▴^___^), JM101[pWK311R] (---◇---) and JM101[pWK311ROmpAd] (^….^▪^….^) are shown. The comparison shows that the bioactivities of the three recombinant bFGF samples and standard bFGF form a superimposed line, supporting that their bioactivities share the same potency.

Purified bFGF from both culture media and lysates of transformants JM101 [pWK3ROmpAd], JM101 [pWK311R] and JM101 [pWK311ROmpAd] were assayed for mitogenic activities performed as described previously^9^. The results revealed that all bFGF samples were as potent as that of the authentic standard (Fig. 3), supporting the fact that bFGF detected in either the cytoplasm or the culture media of all three transformants are fully bioactive.

### Fermentative production of bFGF

Among the four constructs studied, pWK311ROmpAd provided the best performance in bFGF expression (Fig. 2). Therefore, the ability of JM101 [pWK311ROmpAd] to express bFGF using a modified protocol of the fed-batch fermentation process with continuous glucose feeding developed previously for EGF expression^15^ was investigated. However, there were two distinct differences between the current approach and that reported previously^15^. First, IPTG induction was excluded. Second, the duration of feeding was reduced from the formerly reported 9 h^15^ to 6 h in this study. Moreover, after feeding, the cultivation was allowed to continue for 7 more h prior to harvesting (Methods).

**Figure 4:**
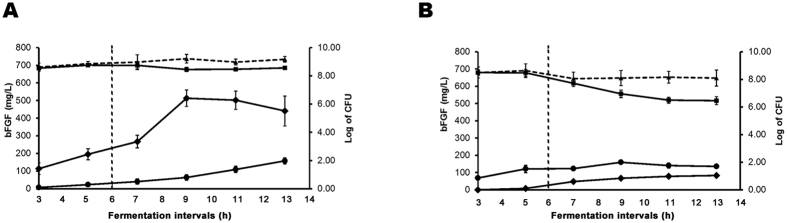
Expression of bFGF by transformants JM101 [pWK311ROmpAd] and JM101 [pWK3R] cultivated in a small-scale fermentor under fed-batch conditions without induction. Culture samples of (**A**) JM101 [pWK311ROmpAd] and (**B**) JM101 [pWK3R] were taken at different time points from the fermentor and viabilities of plasmid-free and plasmid containing cells were determined on plain agar plates (---▴---) and agar plates supplemented with ampicillin (^___^▪^___^), respectively. CFU refers to colony-forming units. Levels of bFGF detected in the cell lysate (^___^♦^___^) and culture supernatant samples (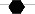) are presented. The fermentation intervals present developments of cell growth and bFGF expression during two different stages of cultivation (partitioned by the vertical dotted line): last phase of glucose feeding (3–6 h) and the “pending period” (6–13 h). Each growth experiment of the two transformants was repeated three times and standard error bars are shown.

**Figure 5:**
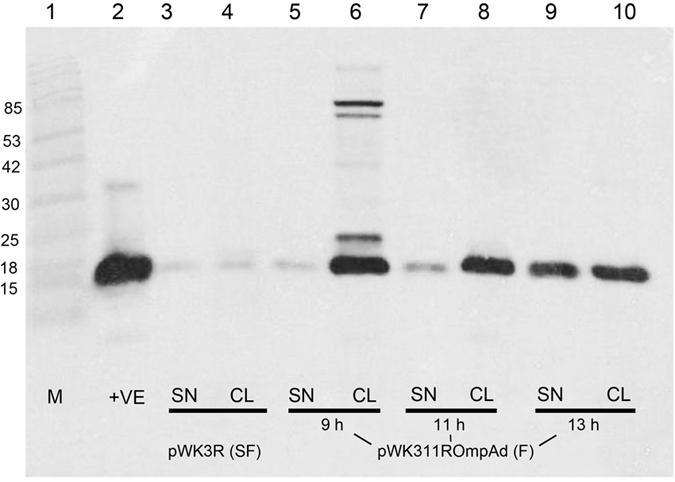
Western blot analysis of bFGF expressed by transformants JM101 [pWK3R] and JM101 [pWK3ROmpAd] grown under shake flask and fermentation conditions. Shake flask (SF) and fermentation (**F**) conditions for growth of JM101 [pWK3R] and JM101 [pWK311ROmpAd], respectively, are described in Methods. Culture supernatant (SN) and cell lysate (CL) samples were prepared from the former (lanes 3–4) and latter (lanes 5–10) cultures and analyzed for bFGF activities by western blotting. The amounts of SN and CL samples loaded were equivalent to 6 μl and 2.5 μl of each culture, respectively. The constructs concerned are indicated beneath the blot. The three time points: 9 h, 11 h and 13 h, at which samples collected from the JM101 [pWK311ROmpAd] culture are denoted. Other symbols used are: M = protein markers (lane 1) in kDa; +VE = bFGF standard (lane 2).

Glucose feeding was commenced at early log-growth phase and was stopped after cell growth had reached the stationary phase, during which production of bFGF was maximum (Fig. 4A) and yields of extracellular bFGF, resulting presumably from minor cell lysis^22^, increased progressively (Figs 4A and 5). During the last 7 h of cultivation, designated the “pending period”, the productivities of bFGF were obviously far better than those detected at earlier time points (Fig. 4A).

Surprisingly, the modified protocol was shown to be highly rewardable to result in a final yield of as high as 610 mg/L of bFGF, e.g., at 5 h after entering the “pending period” (Fig. 4A), which was 1.6 times higher than that derived from the same transformant grown in shake flasks (Fig. 2). The high levels of bFGF expressed did not seem to impose obvious harmful effect on either cell growth or the quality of bFGF, which remained soluble and bioactive (Fig. 3) throughout the fermentation process (Fig. 4A). During the “pending period”, bFGF was progressively released from the lysed cells to the culture medium. The supernatant samples collected at latter time points were shown to provide an impressive source of bFGF, in terms of not only quantity [representing about one-fifth of the overall bFGF yield (Figs 2 and 4A)], but also quality, which contained comparatively “purer” bFGF with lower levels of host cell protein contamination.

Despite employing the refined fermentation protocol, JM101 [pWK3R] (Fig. 4B) did not obtain the same extent of improvement in total bFGF yield as that shown by JM101 [pWK311ROmpAd] (Fig. 4A). Apparently, only one copy of the *bfgf* gene harbored by pWK3R and low cell viabilities of JM101 [pWK3R] (Fig. 4B), which were 100 times lower than those of JM101 [pWK311ROmpAd] at later growth points (Fig. 4A), significantly affected the performance of JM101 [pWK3R] in bFGF expression. Although the highest total yield of JM101 [pWK3R] upon fermentation was over 200 mg/ml (Fig. 4B), which was 2 times higher than the same clone grown in shake flasks (Fig. 2), the level was 3 times lower than that of JM101 [pWK311ROmpAd] grown under the same fermentative conditions (Fig. 4A).

## Discussion

Applying intein-mediated expression systems, we have recently achieved recombinant production of two important skin growth factors, EGF and bFGF, as authentic proteins in *E. coli*^9^. Both EGF^23^,^24^,^25^ and bFGF were identified as precisely processed products detached from their fusion intein partner, VMA, in both the culture medium and the cytoplasm^9^. The same approach has also been employed for co-expression and auto-cleavages of fusion products formed between different inteins and widely dissimilar proteins^11^.

Leveraging the success of the development of the VMA-mediated expression system in producing authentic bFGF^9^, we investigated the effects of two structural modifications: (i) deletion of the *ompA* leader sequence, and (ii) doubling the copy number of the DNA fusion formed between *vma* and the *bfgf* gene, on improving bFGF expression. Apparently, both changes had a positive impact on the production of bFGF (Fig. 2). Moreover, when the two modifications were both introduced into pWK311ROmpAd (Fig. 1), the changes resulted in a much better yield of bFGF (Fig. 2).

The presence of EGF at the N-terminus of the VMA-bFGF fusion appeared to serve as a competent N-extein which facilitated the cleavage between VMA and bFGF^9^. However, fusing OmpA at the N-terminus of VMA-bFGF did not result in a cleavable fusion^9^. It was reasoned that EGF might enable its fusion partner: VMA-bFGF in our previous work^9^,^11^ or VMA-bFGF-VMA-bFGF in this study, to attain an extended and auto-cleavable structure. Presumably, the cleavage sites between VMA and bFGF in the precursor and intermediate products were more exposed and accessible for self-processing.

**Figure 6:**
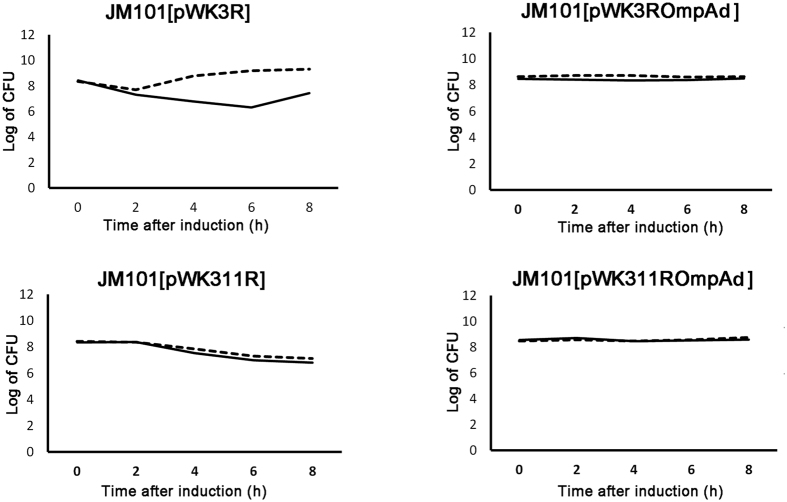
Viable cell counts obtained from the time course study of various *E. coli* cultures. Samples of *E. coli* cultures harboring the four constructs: pWK3R, pWK3ROmpAD, pWK311R and pWK311ROmpAD grown under IPTG induction were taken and assayed for plasmid-free and plasmid containing cells by differential plating. Viable cell counts obtained on plain agar plates (------) and agar plates supplemented with Amp (^_________^) are shown; CFU refers to colony-forming units. The names of the transformants are shown above each graph for indication of the cultures. Each assay was repeated three times (SEM < 0.1). Note that constructs pWK3R and pWK311R were equipped with the *ompA* signal sequence for secretory production of EGF and bFGF, whereas constructs pWK3ROmpAD and pWK311ROmpAD were not. The viable cell counts of the latter two cultures were about 10-fold higher than those of the former ones.

The outcomes of the present study support the following conclusions. First, two copies of the *bfgf* gene yielded better bFGF expression than did one copy. Second, deletion of the *ompA* leader sequence from constructs pWK3ROmpAd and pWK311ROmpAd (Fig. 1) resulted in improved cell viabilities of their transformants in shake flasks (Fig. 6). This notion gained support from the findings that pWK3R, which contained the *ompA* leader sequence (Fig. 1), elicited high plasmid instabilities and low cell viabilities in JM101 [pWK3R] (Fig. 4B), despite the growth of JM101 [pWK3R] carried out under optimized fermentative conditions (Fig. 4B). However, [pWK311ROmpAd], in which *ompA* was deleted, grew with much higher plasmid stabilities and cell viabilities under the same conditions (Fig. 4A). These observations were in line with our previous findings that secretory production of recombinant proteins in *E. coli* would cause detrimental, and even lethal, effects on the host cells^16^,^26^,^27^,^28^,^29^. As expected, deletion of the *ompA* leader sequence from constructs pWK3ROmpAd and pWK311ROmpAd (Fig. 1) resulted in improved cell viabilities of their transformants (Fig. 3). Nonetheless, deletion of the OmpA signal hampered secretion of bFGF, thus resulting in essentially cytoplasmic production of the polypeptide, and hence higher yields of intracellular, or overall, bFGF (Fig. 2). Therefore, possessing both structural modifications, pWK311ROmpAd was revealed to be the most productive construct which was 50–100% more potent than the other three plasmids in bFGF expression (Fig. 2).

The rewarding achievement of pWK311ROmpAd in small scale studies prompted us to investigate its performance in fermentor cultivation. We adapted a glucose feeding fed-batch approach previously employed to yield high levels of EGF in *E. coli*^15^, with the following modifications. First, IPTG induction was excluded in growing JM101 [pWK311ROmpAd] since high efficiencies of transcription of heterologous genes might seriously retard cell growth^16^,^26^,^27^,^28^,^29^. Second, a shortened, 6 h instead of the previously reported 9 h^15^, glucose feeding regime was adopted. Despite using a significantly shorter feeding time, the rate of cell growth was shown to be normal and remained high at a cell density of over 10^8^ cells/ml (Fig. 4A). Obviously, the non-induced conditions offered a favorable instead of a harmful environment for JM101 [pWK311ROmpAd] to grow. Another point supporting this notion was that pWK311ROmpAd was maintained quite stably in its host during growth (Fig. 4A). Otherwise, if bFGF were expressed under IPTG induction, pWK311ROmpAd could have suffered serious instability, as reported previously in studies where other recombinant proteins were expressed under induction^16^,^26^,^27^,^28^,^29^.

Although pWK311ROmpAd was devoid of the *ompA* leader sequence, as a result of cell lysis, high levels of bFGF, reaching 110 mg/L, were released to the culture medium at the latter time points of the “pending period” (Fig. 4A). This concentration, representing one-fifth of the maximum production of bFGF obtainable from JM101 [pWK311ROmpAd], provided a convenient source of less contaminated bFGF. It was previously postulated that high levels of recombinant proteins present in *E. coli* might weaken its membrane structure, thus resulting in increased susceptibility to cell lysis^22^. It will be interesting to explore whether bFGF release may be enhanced with further improvement in the overall expression of the peptide or modulations of the glucose feeding program including the duration of the “pending period”.

The application of the novel approach of gene amplification, as illustrated by the *bfgf/vma*/*bfgf* gene fusion engineered in constructs pWK311R (Fig. 1C) and pWK311ROmpAd (Fig. 1D), is well demonstrated in this report to result in not only better yields but also a homogeneous preparation of bFGF (Table 1). Employing the optimized intein-mediated expression approach, together with a refined, non-induced, fed-batch fermentation protocol, we have been successful in achieving a phenomenal yield of 610 mg/L of bFGF in *E. coli*. Further optimization of both the expression and fermentation conditions may not only enable operations to be performed cost-effectively on a large scale, but may also facilitate efficient production of a wide collection of both intracellular and secretory proteins.

## Methods

### Bacterial strain and chemicals

*E. coli* strain JM101^15^ was the host employed in this study. The Phusion PCR kit, restriction, and modifying enzymes were purchased from New England Biolabs (Ipswich, MA, USA). All oligos were purchased from Invitrogen (Carlsbad, CA, USA). Other chemicals were purchased from Sigma-Aldrich Corporation (St. Louis, MO, USA) unless otherwise specified. Antibodies against bFGF were raised in rabbits.

### Construction of expression constructs

Plasmids pWK311R and pWK3ROmpAd were derived from pWK3R^9^, with the following modifications. The *EcoR*I-*Sph*I fragment of pWK3R was replaced, by a PCR fragment formed using primers P5 – P8 (Table 2), which was composed of the following components: *lac*UV5 promoter, *lac* operator (*lac*O), ribosomal binding site (RBS), and *egf* gene to form pWK3ROmpAd. Another PCR fragment, Fragment A, synthesized using primers P1–P4 (Table 2), containing the *bfgf* gene fused with the VMA coding sequence (*vma*), was inserted into the *Bam*HI site of pWK3R to form pWK311R. Lastly, to develop pWK311ROmpAd, Fragment A was inserted into the *Bam*HI site of pWK3ROmpAd.

### Shake flask cultivations

*E. coli* transformants were grown at 34 °C in MMBL medium^9^ supplemented with 70 μg/ ml of ampicillin. In time-course experiments, a 250 ml flask containing 50 ml of growth medium was inoculated with a freshly grown colony and shaken at 250 rpm and 34 °C until the culture reached an A_550_ reading of 8.0. Subsequently, a final concentration of 0.1 mM IPTG was added and the culture was continuously grown for 8 h. Then 1 ml of the culture was centrifuged and the SN was saved. The cell pellet was suspended in 120 μl of Tris.HCl buffer (50 mM, pH 8.0), followed by an addition of 83 μl of EDTA solution (0.25 M, pH 8.0). The cell mixture was incubated on ice for 5 min and then treated with 120 μl of lysozyme solution (10 mg/ml) at 37 °C for 20 min. After addition of 83 μl of lysis buffer (10 mM EDTA, 10% Triton X-100, and 50 mM Tris.HCl, pH 8.0), the tube was inverted gently, followed by spinning at 13,000 rpm for 10 min to remove the cell debris. Both the clarified lysate (CL) and culture supernatant (SN) fractions were analyzed for bFGF by Western blot analysis, of which the images were quantified by densitometry using the ImageJ software (National Institutes of Health, USA) as reported previously^9^.

### Purification and analysis of bFGF

The purification of bFGF using heparin-agarose chromatography and analysis of the purified bFGF by liquid chromatography tandem mass spectrometry were described previously^9^.

### Biological assays of bFGF

The mitogenic effects of purified bFGF samples on the proliferation of BALB/C 3T3 fibroblast cells were analyzed by the MTT assay as described previously^9^.

### Fermentation

MMBL medium^9^ was used throughout the entire fermentation process including the preparation of starter cultures. To begin with, a fresh colony of JM101 [pWK311ROmpAd] or JM101 [pWK3R] was inoculated in 50 ml of MMBL medium supplemented with 70 μg/ml of ampicillin. The cells were grown at 34 °C until an A_550_ reading reached 2.0. Then 15 ml of the starter were added into 135 ml of fresh MMBL medium supplemented with 70 μg/ml of ampicillin, and the culture was grown for 3 h at 34 °C. The entire 150 ml culture was then added into a 2 L fermentor containing 1.35 L of fresh MMBL medium. The pH of the culture was maintained at 6.8 using 1 M NaOH solution. When the pH began to decrease, the culture was fed with 50% glucose at a rate of 4 ml/h. The pH was maintained at 6.8 until the A_550_ reading was 15.0, which took about 6 h to reach. The feeding process was then stopped, but the operation of the fermentor was allowed to continue for the next 7 h (“pending period”), during which lytic release of bFGF into the medium was expected to occur. Culture samples were collected at different time points of the fermentation process. The fractionated cell pellet and culture supernatant samples were then subjected to various analyses as discussed in Results above.

## Additional Information

**How to cite this article**: Kwong, K. W. Y. *et al.* Intein mediated hyper-production of authentic human basic fibroblast growth factor in *Escherichia coli. Sci. Rep.* **6**, 33948; doi: 10.1038/srep33948 (2016).

## Figures and Tables

**Table 1:** Analysis of bFGF, purified from the cell samples of *E. coli* transformants harboring the four plasmid constructs/a, by liquid chromatography tandem mass spectrometry.

Peptide^b^,^c^	Mr(Calc)^d^	Mr(Expt)^e^
(1) ^NH2-^PALPEDGGSG^|10^AFPPGHFK	1779	1779
(2) RLYCKNGGF^|30^FLR	1529	1530
(3) NGGF^|30^FLR	809	808
(4) IHPDGRV^|40^DGVR	1219	1220
(5) EKSDPH^|50^IK	952	952
(6) SDPHIKLQLQAEER^|60^	1662	1663
(7) ^|60^GVVSIKGVCA^|70^NR	1259	1258
(8) YLAMKED^|80^GR	1081	1082
(9) CVT^|90^DECFFFER	1509	1508
(10) LE^|100^SNNYNTYR	1273	1272
(11) LESNNYNTYR	1272	1273
(12) ^|110^KYTSWYVALK^|120^	1259	1258
(13) TGQYKLGSK^|130^TGPGQK	1548	1548
(14) AILFL^|140^PMSAK	1090	1089
(15) ^|130^TGPGQKAILFL^|140^PMSAKS	1744	1744
(16) AILFL^|140^PMSAKS^-COOH^	1176	1177

^a^The samples included cell lysates and culture media of both shake flask and fermentative cultures. All four transformants: JM101[pWK3R], JM101[pWK3ROmpAd], JM101[pWK311R] and JM101[pWK311ROmpAd], were included in shake flask cultures, whereas only JM101[pWK3R] and JM101[pWK311ROmpAd] were also involved in fermentor cultivation.

^b^Subsequent to trypsin digestion of purified bFGF, a total of 466 peptides were identified by the Mascot search engine.

^c^The availability of mature bFGF sequence in the literature has facilitated the selection and alignment of sequencing results of the trypsin digested peptides (16 of them as revealed in the above Table) to finally obtain a full sequence of the recombinant bFGF as shown below. NH_2_PALPEDGGSG^|10^AFPPGHFKDP^|20^KRLYCKNGGF^|30^FLRIHPDGRV^|40^DGVREKSDPH^|50^IKLQLQAEER^|60^GVVSIKGVCA^|70^NRYLAMKED^|80^GRLLASKCVT^|90^DECFFFERLE^|100^SNNYNTYRSR^|110^KYTSWYVALK^|120^RTGQYKLGSK^|130^TGPGQKAILFL^|140^PMSAKS-COOH.

^d^Theoretical mass-to-charge ratio of the peptide.

^e^The experimental mass-to-charge ratio of the peptide.

**Table 2:** Oligos used in the study.

Primer	Sequence^a^
P1	5′-CACTGAAACGCACTGGGCAG-*TATAAACTTGGATCCAA*-3′
P2	5′-ACCCTTGGCAAAGCAGCTCTTAGCAGACAT-*TATAAACTTGGATCCAAAACAG* -3′
P3	5′-ATGTCTGCTAAGAGCTGCTTTGCCAAGGGT-3′
P4	5′-TTTCTGCCCAGGTCCTGTTT*T-GGATCCAAGTT*-3′
P5	5′-ACGAGGCCCTTTCGTCTTCA-*AGAATTCGCAT*-3′
P6	5′-CAGAGTCACTATTCATAATTTTTTC-3′
P7	5′-GAAAAAATTATGAATAGTGACTCTG-3′
P8	5′-CAACAACACAGTTGCATGCATACTT-3′

^a^Oligo sequences presented in italics are *bfgf* unrelated sequences containing restriction sites downstream from the designed PCR primers. Restriction sites on the italic sequences are underlined.
